# Intraoperative or postoperative stereotactic radiotherapy for brain metastases: time to systemic treatment onset and other patient-relevant outcomes

**DOI:** 10.1007/s11060-023-04464-7

**Published:** 2023-10-09

**Authors:** Cas S. Dejonckheere, Julian P. Layer, Motaz Hamed, Katharina Layer, Andrea Glasmacher, Lea L. Friker, Anna-Laura Potthoff, Thomas Zeyen, Davide Scafa, David Koch, Stephan Garbe, Jasmin A. Holz, Fabian Kugel, Molina Grimmer, Frederic Carsten Schmeel, Gerrit H. Gielen, Helmut Forstbauer, Hartmut Vatter, Ulrich Herrlinger, Frank A. Giordano, Matthias Schneider, Leonard Christopher Schmeel, Gustavo R. Sarria

**Affiliations:** 1 Department of Radiation Oncology, University Hospital Bonn, Venusberg-Campus 1, 53127 Bonn, Germany; 2 Institute of Experimental Oncology, University Hospital Bonn, 53127 Bonn, Germany; 3 Department of Neurosurgery, University Hospital Bonn, 53127 Bonn, Germany; 4 Institute of Neuropathology, University Hospital Bonn, 53127 Bonn, Germany; 5 Division of Clinical Neuro-Oncology, Department of Neurology, University Hospital Bonn, 53127 Bonn, Germany; 6 Department of Neuroradiology, University Hospital Bonn, 53127 Bonn, Germany; 7 Oncology Practice Network Troisdorf, 53840 Troisdorf, Germany; 8 Department of Radiation Oncology, University Medical Center Mannheim, 68167 Mannheim, Germany

**Keywords:** Intraoperative radiotherapy, Brain metastasis, Stereotactic radiotherapy, Time to next treatment

## Abstract

**Purpose:**

Intraoperative radiotherapy (IORT) has become a viable treatment option for resectable brain metastases (BMs). As data on local control and radiation necrosis rates are maturing, we focus on meaningful secondary endpoints such as time to next treatment (TTNT), duration of postoperative corticosteroid treatment, and in-hospital time.

**Methods:**

Patients prospectively recruited within an IORT study registry between November 2020 and June 2023 were compared with consecutive patients receiving adjuvant stereotactic radiotherapy (SRT) of the resection cavity within the same time frame. TTNT was defined as the number of days between BM resection and start of the next extracranial oncological therapy (systemic treatment, surgery, or radiotherapy) for each of the groups.

**Results:**

Of 95 BM patients screened, IORT was feasible in 84 cases (88%) and ultimately performed in 64 (67%). The control collective consisted of 53 SRT patients. There were no relevant differences in clinical baseline features. Mean TTNT (range) was 36 (9 − 94) days for IORT patients versus 52 (11 − 126) days for SRT patients (*p* = 0.01). Mean duration of postoperative corticosteroid treatment was similar (8 days; *p* = 0.83), as was mean postoperative in-hospital time (11 versus 12 days; *p* = 0.97). Mean total in-hospital time for BM treatment (in- and out-patient days) was 11 days for IORT versus 19 days for SRT patients (*p* < 0.001).

**Conclusion:**

IORT for BMs results in faster completion of interdisciplinary treatment when compared to adjuvant SRT, without increasing corticosteroid intake or prolonging in-hospital times. A randomised phase III trial will determine the clinical effects of shorter TTNT.

## Introduction

Following recent advances in systemic treatment options and subsequent improved overall survival, the relative diagnostic incidence of brain metastases (BMs) is on the rise [1–3]. A cornerstone in their managment is postoperative local control, as this remains the primary treatment objective to prevent neurological decline and avoid additional interventions [4]. For large or symptomatic lesions, the standard of care includes maximal surgical resection followed by one to several fractions of adjuvant stereotactic radiotherapy (SRT), in order to improve local control. The latter yields superior outcomes over whole-brain radiotherapy in terms of neurocognition and quality of life [5–7]. To allow for postoperative patient stabilisation and sufficient surgical wound healing, adjuvant radiation treatment is only initiated after a postoperative interval of several weeks, which increases the overall BM treatment time and delays the onset of systemic treatments [8].

In recent years, intraoperative radiotherapy (IORT) is emerging as a viable alternative treatment option for resectable BMs [9–11]. Low-level X-rays applied directly to the resection cavity result in high rates of local tumour control, while simultaneously omitting the need for adjuvant SRT in the case of solitary BMs or reducing the total number of treatment days in the case of multiple BMs [12–15]. A swift completion of interventional BM treatments might shorten the time to systemic therapy initiation, which could potentially improve survival outcomes, especially in treatment-naive patients or those with high tumour burden at the time of BM surgery. Furthermore, the instant application of a single high local radiation dose might prevent early repopulation of residual microscopic tumour. Other advantages of IORT include a steep dose gradient with improved sparing of healthy brain tissue and omitting challenging target volume delineation caused by postoperative tissue alterations [16]. Despite all the above-mentioned, patient-centered outcomes are currently scarce in this setting. Herein, we report meaningful secondary endpoints of IORT patients compared to an institutional SRT cohort, including time to next extracranial oncological treatment (TTNT), duration of postoperative corticosteroid treatment and in-hospital times.

## Materials and methods

### IORT patients

Consecutive patients who underwent BM exeresis combined with IORT at our university center within a prospective registry between November 2020 and June 2023 were screened. Criteria for surgery included presence or severe risk of acute neurological impairment and clinically significant mass effect, i.e. signs of raised intracranial pressure or hemispheric shift. In patients with multiple BMs, only the clinically manifest lesion was considered for surgical removal in order to prevent mass effects or tumour-related hydrocephalus. IORT was considered in the case of planned gross total resection and intraoperative neuropathological confirmation of BM by frozen section.

Preoperative contrast-enhanced T1-weighted magnetic resonance imaging (MRI) provided three-dimensional guidance for both surgery and IORT. Optic nerves, optic chiasm, and brainstem were identified pre- and intraoperatively as organs at risk (OARs). A spherical applicator ranging from 1.5 to 5 cm diameter was placed into the surgical cavity according to the best-fit rule, covering the entire surface. A standard recommended dose of 30 Gy was prescribed to the applicator surface (nominal 50 kV photons) [16]. Delivered OAR doses were calculated based on dose-depth template profiles corresponding to the applicator diameter. In the case of OAR doses exceeding Quantitative Analyses of Normal Tissue Effects in the Clinic (QUANTEC) constraints (i.e. 12 Gy for the optical system or 12.5 Gy for the brainstem), a decrease in the prescribed dose to 16 Gy (minimum) was acceptable. IORT was delivered with the INTRABEAM 600 (Carl Zeiss Meditec AG, Oberkochen, Germany).

Patient demographics and clinical characteristics were collected from the electronic health records. The Karnofsky Performance Score (KPS) classified patients according to their functional status at the time of admission, with a stratification cut-off of 70, depending on a patient’s ability to carry out normal activity and work [17]. Diagnosis-Specific Graded Prognostic Assessment (DS-GPA) scores were calculated by standard procedures [18].

This study was conducted in accordance with the Declaration of Helsinki and approved by the Institutional Review Board of the University Hospital Bonn (018/21 and 057/22).

### Controls

The control collective consisted of two patient groups: patients screened for IORT but not receiving it for one of several reasons (Table 1), thus subsequently requiring adjuvant SRT of the resection cavity and patients planned for adjuvant SRT of the resection cavity within the same time frame. Inclusion criteria for both groups were surgically resected histologically confirmed solid tumour BM receiving one to seven fractions of adjuvant SRT of the resection cavity and a total BM number ≤ 10 at the time of surgery.

**Table 1:** Reasons for not receiving IORT for resectable brain metastasis. IORT = intraoperative radiotherapy; OAR = organ at risk

**IORT not possible or feasible** (***n*** **= 11)**
logistics (*n* = 6)
expected violation of OAR constraint (*n* = 3)
patient declining surgery (*n* = 2)
**IORT possible and feasible, but not performed (*n*** **= 20)**
frozen section unclear (*n* = 7)
resection cavity not spherical (*n* = 5)
technical reasons (*n* = 4)
logistics (*n* = 2)
measured violation of OAR constraint (*n* = 1)
patient declining surgery (*n* = 1)

All patients received a planning computer tomography (CT) in supine position with an individual thermoplastic stereotactic fixation mask. A postoperative contrast-enhanced T1-weighted planning MRI with 1 mm slice thickness was coregistered with this planning CT and the gross tumour volume (GTV) was defined as the resection cavity including any possible residual contrast (Gd) enhancement. A 2 mm isotropic margin was added for the planning target volume (PTV), as per institutional standards. SRT was administered with intensity-modulated image-guided techniques, employing 6 − 10 MV photon energies and ensuring a target volume coverage of 99 − 120%. All patients were treated on a TrueBeam STx (Varian Medical Systems, Palo Alto, CA, USA) linear accelerator, using ExacTrac (Brainlab, München, Germany) for position matching.

### Literature search

To put the data into perspective, international literature (MEDLINE) and study registries (National Clinical Trials) were screened for similar retrospective and prospective IORT collectives, using the search terms *intraoperative radiotherapy* and *brain metastasis*. Where available, data on TTNT were extracted and summarised.

### Statistical analysis

The primary endpoint of this trial was TTNT, defined as the number of days between BM resection and start of the next oncological intervention (systemic treatment, extracranial surgery or radiation) for each of the groups. Patients were included in the analysis if they received such treatment and the exact date of treatment start was known. Reasons for exclusion were initial patient decline of the proposed subsequent treatment and logistic reasons for delay in case of extracranial surgery.

Mean, median, standard deviation (SD), and range were calculated for all applicable clinical data. Differences in baseline patient characteristics between groups were assessed using Fisher’s exact test, Pearson’s *χ*^2^, or Student’s unpaired *t*-test, as appropriate. For the comparison of TTNT between groups, the Mann-Whitney-*U*-test was used. The log-rank test was used for the statistical assessment of event rates, presented according to the Kaplan-Meier method. The statistical significance level was defined as *p* < 0.05. Microsoft Excel version 16 (Microsoft, Redmond, WA, USA), SPSS Statistics version 27 (IBM, Armonk, NY, USA), and GraphPad Prism version 9 (GraphPad Software, San Diego, CA, USA) were used to perform the analyses and Adobe Illustrator 2023 (Adobe Inc., Mountain View, CA, USA) to generate graphical images.

## Results

**Fig. 1:**
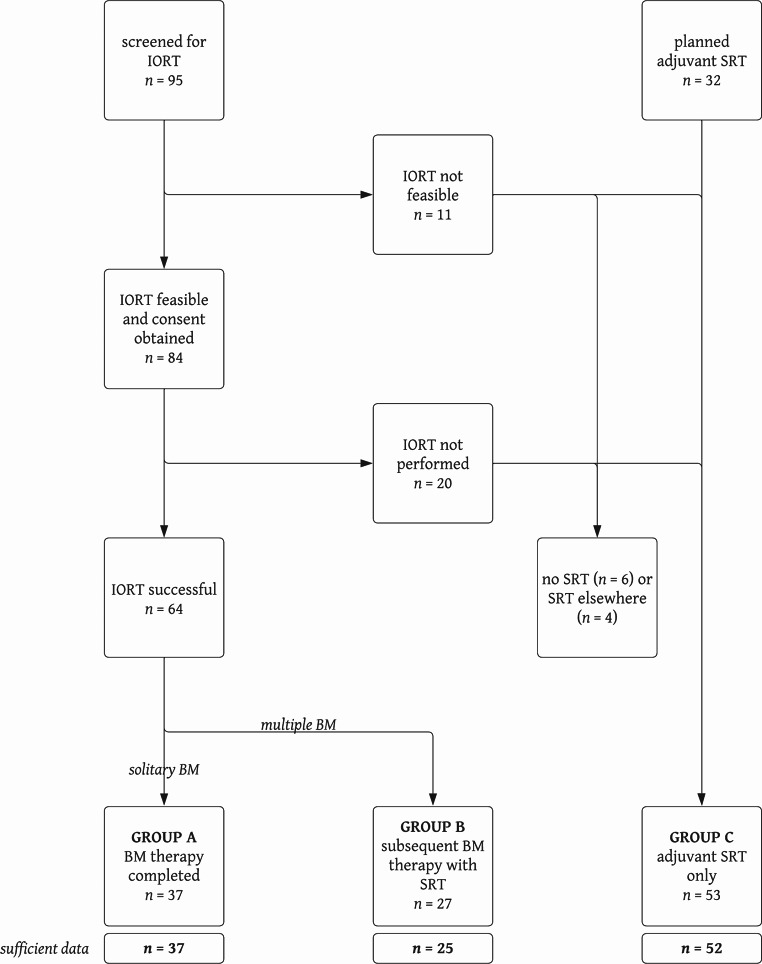
Flowchart of patient selection. IORT = intraoperative radiotherapy; SRT = stereotactic radiotherapy; BM = brain metastasis

Of 95 BM patients screened, IORT was deemed feasible in 84 cases (88%) and ultimately performed in 64 (67%). Sufficient data were available for 62 patients undergoing IORT and 52 receiving adjuvant SRT of the resection cavity. A flowchart of patient selection is provided in Fig. 1. Patient and treatment characteristics are summarised in Table 2. There were no relevant differences in baseline characteristics between both groups. Data on local control, distant brain failure, radiation necrosis incidence, and overall survival for a subset of 35 IORT patients with mature follow-up are reported elsewhere [15].

**Table 2:** Patient and treatment characteristics. IORT = intraoperative radiotherapy; SRT = stereotactic radiotherapy; KPS = Karnofsky Performance Score; DS-GPA = Diagnosis-Specific Graded Prognostic Assessment; Gy = Gray; GI = gastrointestinal; GU = genitourinary; BM = brain metastasis

	IORT	SRT	*p*
*n*	62	52	
female sex, *n* (%)	31 (50)	25 (48)	0.85
median age at surgery (range) in years	63 (35 − 91)	64 (34 − 87)	0.86
KPS at surgery, *n* (%) ≥ 70 < 70	49 (79) 13 (21)	43 (83) 9 (17)	0.77
median DS-GPA at surgery (range)	2 (0 − 4)	2 (0 − 4)	0.39
extracranial metastases at surgery, *n* (%)	52 (84)	27 (68)	0.09
median radiation dose (range) in Gy *	30 (16 − 30)	35 (20 − 45)	
primary lobe, *n* (%)			
frontal parietal occipital temporal cerebellum	24 (39) 2 (3) 16 (26) 8 (13) 12 (19)	17 (33) 10 (19) 8 (15) 8 (15) 9 (17)	0.07
primary tumour, *n* (%)			
lung melanoma GI GU breast gynaecological other	37 (60) 10 (16) 5 (8) 6 (10) 3 (5) 1 (2) 0 (0)	25 (48) 9 (17) 7 (13) 3 (6) 3 (6) 1 (2) 4 (8)	0.26
number of BMs at surgery, *n* (%)			
solitary multiple (range of multiple BMs)	36 (58) 26 (42) (2 − 10)	31 (60) 21 (40) (2 − 10)	0.49
median time (range) to			
SRT onset in days SRT completion in days		25 (11 − 173) 34 (11 − 187)	

* Due to radiobiological differences between IORT and SRT, the difference between the administered doses is not deemed relevant and thus not calculated

**Fig. 2:**
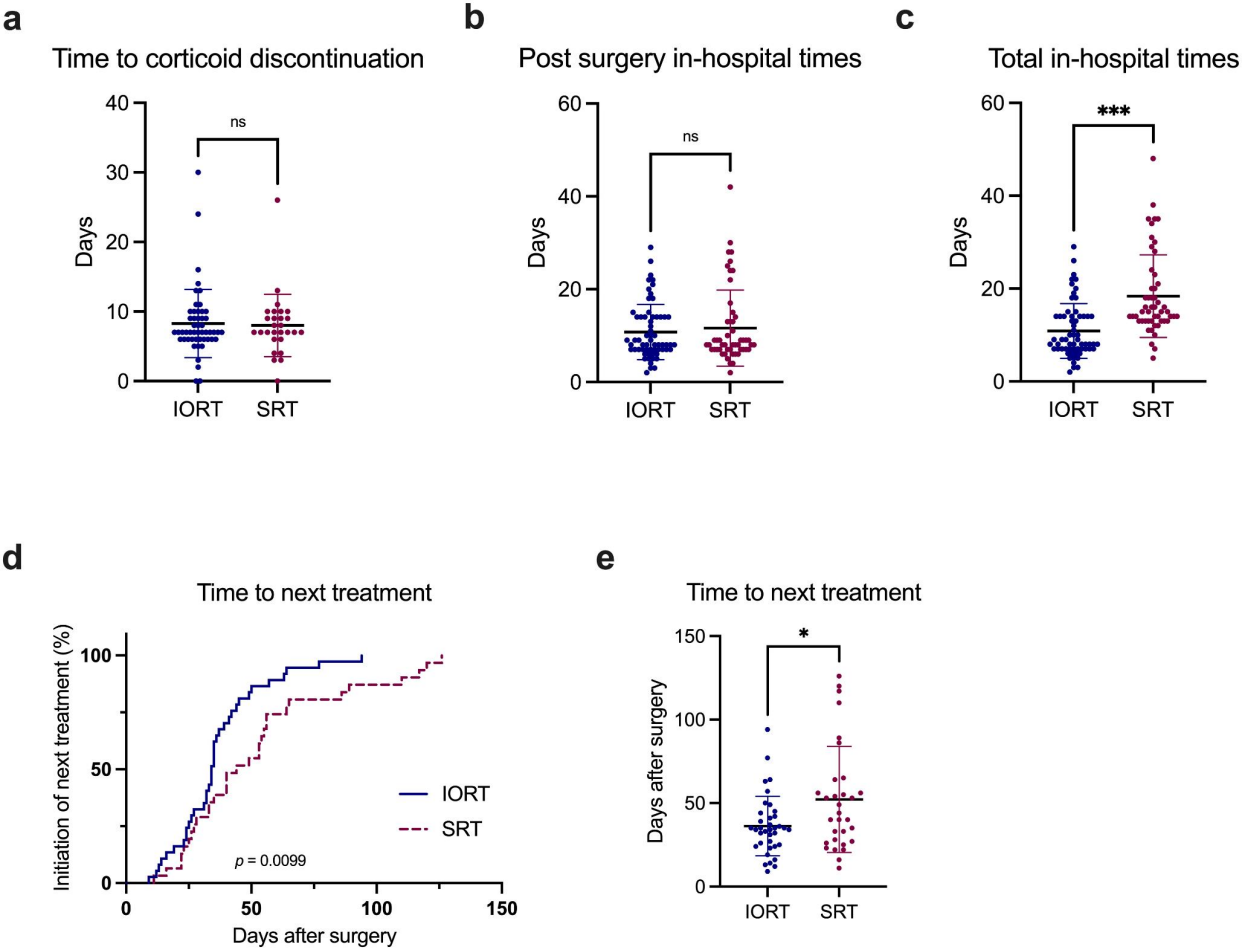
Comparison of meaningful patient-centered secondary endpoints between IORT and adjuvant SRT patients. Scatter plots showing (**a**) time in days to corticoid discontinuation, (**b**) postoperative in-patient time, and (**c**) total (in- and out-patient) in-hospital time, Mann-Whitney-*U*-test. (**d**) Kaplan-Meier curve for patients reaching initiation of their next extracranial oncological treatment (log-rank test). (**e**) Scatter plot for time to next extracranial oncological treatment (Mann-Whitney-*U*-test). * *p* < 0.05, *** *p* < 0.001, ns = not significant. IORT = intraoperative radiotherapy; SRT = stereotactic radiotherapy

Thirty-nine patients (63%) in the IORT group versus 31 patients (60%) in the adjuvant SRT group received postoperative extracranial treatment, with systemic therapies being the most common (84% and 97%, respectively). There was no difference in the types of additional treatment (*p* = 0.11). The location (i.e. same or different center), a potential confounder which can cause logistic delay due to outpatient referral systems, was not significantly different between both groups (*p* = 0.08). Neither duration of postoperative corticosteroid treatment nor postoperative in-hospital time was significantly different between both groups: *p* = 0.83 (Fig. 2a) and *p* = 0.97 (Fig. 2b), respectively. Mean total in-hospital time for BM treatment (in- and out-patient) was 11 days for IORT versus 19 days for SRT patients (*p* < 0.001; Fig. 2c). Mean TTNT (range) was 36 (9 − 94) days for IORT patients versus 52 (11 − 126) days for adjuvant SRT patients (*p* = 0.01; Fig. 2d *− e*). Results are summarised in Table 3.

**Table 3:** Time to next treatment, duration of postoperative corticosteroid treatment, and in-hospital time. IORT = intraoperative radiotherapy; SRT = stereotactic radiotherapy; SD = standard deviation

	IORT	SRT	*p*
received postoperative extracranial treatment, *n* (%)			
yes no unknown	39 (63) * 18 (29) ** 5 (8)	31 (60) 15 (29) 6 (11)	0.99
type of additional treatment, *n* (%)			
chemotherapy immunotherapy chemoimmunotherapy antihormone therapy extracranial surgery extracranial radiotherapy	6 (16) 20 (54) 4 (11) 1 (3) 3 (8) 3 (8)	12 (39) 12 (39) 6 (19) 0 (0) 1 (3) 0 (0)	0.11
location of postoperative extracranial treatment, *n* (%)			
same center different center	21 (54) 18 (46)	23 (74) 8 (26)	0.08
time to next treatment			
median (range) in days mean ± SD in days	34 (9 − 94) 36 ± 18	44 (11 − 126) 52 ± 32	**0.01**
postoperative corticosteroid treatment			
median (range) in days mean ± SD in days	7 (0 − 30) 8 ± 5	7 (0 − 14) 8 ± 3	0.83
postoperative in-hospital time (in-patient)			
median (range) in days mean ± SD in days	8 (2 − 29) 11 ± 6	8 (2 − 42) 12 ± 8	0.97
total in-hospital time (in- and out-patient)			
median (range) in days mean ± SD in days	8 (2 − 29) 11 ± 6	15 (7 − 48) 19 ± 9	**< 0.001**

* Extracranial surgery had to be postponed in one patient suffering COVID pneumonia and another patient initially declined immunotherapy. Both patients were excluded from the analysis

** Two patients had already started systemic therapy prior to surgery and were also excluded from the analysis

**Table 4:** Other published IORT collectives. IORT = intraoperative radiotherapy; TTNT = time to next treatment; 1yLCR = 1-year local control rate; 1yDBC = 1-year distant brain control; RN = radiation necrosis; 1yOS = 1-year overall survival; SRT = stereotactic radiotherapy: CI = confidence interval; n.r. = not reported

author (year)	type	IORT outcome of published trials				*n*		TTNT (days)
		1yLCR (%)	1yDBC (%)	RN (%)	1yOS (%)	IORT	SRT	
current (2023)	prospective study registry	97 *	74 *	3 *	58 *	62	52	*mean (range)* IORT: 36 (9 − 94) SRT: 52 (11 − 126)
Brehmer et al. (2023) [19]	prospective phase II (preliminary)					35	/	*mean (95% CI)* 45 (35–55)
Guedes de Castro et al. (2023) [23]	prospective phase II	88	13	10	80	10	/	n.r.
Diehl et al. (2022) [14]	retrospective	93	71	11	58	18	/	in 5 IORT patients ≤ 15 (shorter than wound healing and adjuvant SRT would have required)
Kahl et al. (2021) [13]	retrospective	84	34	3	62	40	/	*median (range)* 18 (0–130)
Cifarelli et al. (2019) [12]	retrospective	88	58	7	73	54	/	n.r.
Brehmer et al. (2018) [22]	prospective phase II (preliminary)					10	19	*mean (range)* IORT: 46 (27–83) SRT: 61 (16–229)
Weil et al. (2015) [24]	prospective	n.r.	n.r.	13	n.r.	23	/	n.r.

* Results of a subset of 35 IORT patients with mature follow-up [15]

## Discussion

IORT for resectable BM yields comparable outcome to adjuvant SRT of the resection cavity in terms of local control and radiation necrosis rates [12–15, 19]. As long-term follow-up results from the first prospective IORT collectives are maturing, we here focus on meaningful secondary endpoints that have a major impact on treatment decisions, both from the patients’ but also from an economical and logistical perspective. The high incidence of BMs along with their generally poor prognosis indicate that every treatment step should be optimised. Asymptomatic patients might be diagnosed during staging of an extracranial primary tumour, meaning that in such treatment-naive patients, rapid completion of interdisciplinary BM treatment is of particularly high interest, as it might shorten the time to subsequent salvage therapy, which could potentially impact survival chances.

This is the first assessment of IORT feasibility in daily practice of a specialised university center with high turnover, demonstrating a feasibility rate of 88%, which proves the general applicability of IORT as a standard procedure for BM treatment. In the comparative analysis, extracranial oncological therapy could be started on average 16 days earlier following IORT, regardless of potential confounders such as type of treatment or location. If IORT is not available or possible, standard SRT of the resection cavity should be performed to improve local control [5, 6]. In order to prevent impaired surgical wound healing, adjuvant SRT is initiated after a postoperative interval of several weeks, which increases overall BM treatment time and delays onset of salvage systemic therapy. Yaghi et al. (*n* = 176) found that a postoperative delay of > 22 days had a decreased risk of all-cause mortality [8]. However, those waiting > 40 days after BM resection doubled their risk of local tumour progression. The median postoperative time to SRT onset was 25 days in the current SRT collective, well within this time frame.

Reasons for longer TTNT in SRT patients are many fold and include incomplete staging, which might be postponed until after SRT (e.g. due to conflicting appointments), side effects, undesirable combination with planned systemic therapy (e.g. BRAF and MEK inhibitors in melanoma patients), patient refusal to undergo parallel treatments [20]. In line with our previous report on posteroperative morbidity, we demonstrate that IORT is not associated with prolonged hospitalisation or corticosteroid intake [21]. Moreover, patients who underwent IORT had a faster completion of interdisciplinary BM treatment, when compared to those undergoing adjuvant SRT. Even though postoperative in-hospital times were similar, total in-hospital times (in- and out-patient) for BM treatment were significantly shorter for IORT patients (8 days on average), which might save limited treatment resources, reduce BM treatment costs, and positively impact quality of life.

Apart from shorter TTNT and faster recovery after BM treatment, IORT has several other theoretical advantages. The instant application of a single high local radiation dose might prevent early repopulation of residual microscopic tumour and the steep dose gradient improves sparing of healthy brain tissue, potentially preserving neurological functions and possibly improving subsequent re-irradiation options if ever needed. In patients with solitary BM, a repeat MRI for treatment planning is not required, which further reduces costs and in-hospital time. With IORT, challenging target volume delineation caused by postoperative tissue alterations can be omitted. Furthermore, completing radiation treatment while the patient is asleep promotes comfort and reduces patient burden. There is, however, a general lack of evidence on these theoretical advantages of IORT, which is why they should be assessed in ongoing and future prospective trials.

SRT does have the advantage that dose distribution and OAR constraints can be reproduced more accurately, for example in patients requiring SRT for other BMs at a later point in time. An ongoing trial of image-guided IORT will enable real-time planning.

Other published IORT collectives were identified, the results of which are summarised in Table 4. Only Brehmer et al. directly compared TTNT between their prospectively recruited IORT patients (*n* = 10) and a control collective of patients undergoing adjuvant SRT of the resection cavity within the same time frame (*n* = 19) [22]. On average, IORT patients started systemic treatment 15 days earlier when compared to those receiving adjuvant SRT, in accordance with our results. Mean postoperative time to SRT onset was 27 days, consistent with the recommendation of Yaghi et al. As this was a planned safety interim analysis, it was underpowered for this secondary endpoint, but the preliminary data confirm that TTNT tends to be reduced in IORT patients. The prospective phase II trial is designed to recruit 50 patients, will evaluate local efficacy of IORT for BM, and will assess TTNT as a preplanned secondary endpoint (INTRAMET; NCT03226483) [19]. Kahl et al. reported a median TTNT (range) of 18 (0–130) days after IORT in 24 patients requiring subsequent systemic treatment [13]. Diehl et al. observed that in 5 IORT patients, TTNT was ≤ 15 days, which is shorter than wound healing and adjuvant SRT would have required [14]. Both retrospective cohorts did, however, not include a control collective receiving adjuvant SRT of the resection cavity.

This trial carries certain limitations. First, a relatively small sample size, which might be subject to selection bias. Due to the recent implementation of IORT in clinical BM workflows, there are still limited available data on this topic. To our best knowledge, we present the largest collective to date investigating TTNT in this context. Secondly, BM patients represent a heterogeneous collective, with a multitude of systemic treatment options and required diagnostic investigations. The latter could have led to differences in TTNT between IORT and SRT patients. The similarity of baseline patient and treatment characteristics does, however, partly compensate for this. Lastly, it cannot be yet concluded if shorter TTNT and faster completion of BM treatment translate into improved survival or quality of life. As data of prospective IORT trials are maturing, the clinical relevance of these parameters will be elucidated.

## Conclusion

IORT for BMs results in faster completion of interdisciplinary treatment when compared to adjuvant SRT, without increasing corticosteroid intake or prolonging hospital stay. Apart from emerging evidence regarding excellent local control and comparable radiation necrosis rates, these data add to the favourable therapy profile of IORT in this setting. A randomised phase III trial will determine the clinical effects of shorter TTNT.

## Data Availability

Data will be made available upon reasonable request to the corresponding author.
